# Peripheral Facial Palsy in Emergency Department

**Published:** 2018-05

**Authors:** José Ferreira-Penêda, Raquel Robles, Isabel Gomes-Pinto, Pedro Valente, Nuno Barros-Lima, Artur Condé

**Affiliations:** 1 *Department of Otorhinolaryngology, Centro Hospitalar Vila Nova de Gaia, Espinho, Portugal.*

**Keywords:** Bell Palsy, Herpes simplex, Otorhinolaryngologic disease, Peripheral Facial paralyses

## Abstract

**Introduction:**

Peripheral facial palsy (PFP) is commonly diagnosed in every emergency department. Despite being a benign condition in most cases, PFP causes loss in quality of life mostly due to facial dysmorphia. The etiology of PFP remains unknown in most cases, while medical opinion on epidemiology, risk factors and optimal treatment is not consensual. The aim of this study was to review the demographic characteristics of our patients and the medical care administered in our emergency department.

**Materials and Methods:**

Emergency episodes occurring in a 4-year period and codified as facial nerve pathology were analyzed. IBM SPSS software was used for statistical analysis.

**Results:**

In total, 582 emergency episodes were obtained. Due to inexpressive representation of other causes of PFP in our study, we focused our analyses on the 495 patients who were considered to have idiopathic PFP. There was equal distribution among genders, and all age ranges were affected. There were no clear epidemic phenomena. Hypertension was not a statistically significant risk factor for Bell's palsy. Most patients sought medical care in the early stages of the disease and complained of isolated facial weakness. Most patients had mild-to-moderate symptoms. Previous upper way infections (PUAI) were more frequent among children. There was a statistically significant difference regarding computed tomography (CT) scan requests among specialties.

**Conclusion:**

Epidemiologic findings were consistent with most literature on Bell's palsy. Drug therapy is widely used and follows current guidelines. The role of PUAI in the pediatric population must be investigated. Despite evidence of good medical practice, there was an excess of CT scans requested by physicians other than otorhinolaryngologists.

## Introduction

Peripheric Facial Palsy (PFP) is the most prominent manifestation of facial nerve disorders (1). Facial motor weakness is variable and may be accompanied by others symptoms such as dysgeusia, xerostomia, altered facial sensation, vestibular dysfunction and pharyngeal paranesthesia, due not only to parasympathetic and special sensory functions of the facial nerve, but also due to multiple connections with other cranial nerves (2–4). PFP may be congenital (genetic determined or birth related) or acquired (1,2). Acquired conditions may be traumatic (surgical, head trauma), infective (varicella zoster virus, otitis media), inflammatory (autoimmune disease, sarcoidosis), neurological (multiple sclerosis, Guillain-Barré syndrome) or idiopathic (Bell’s palsy) (1,2). Up to 70% of the cases are labeled as idiopathic (4–6), and for this reason Bell’s palsy deserves special attention.

Known for centuries, even before Charles Bell’s famous description (2), Bell’s palsy affects both genders and both sides of the face in equal proportions (2,3). It is present across all ages, but a higher incidence is noted between the ages of 15 and 45 years (2,5,7). The reported incidence is fairly constant among studies, ranging from 11 to 40.2/100,000 (2,8). The risk of Bell’s palsy is higher during pregnancy, in hypertensive, diabetic or immunocompromised patients, and following viral upper respiratory infections (2,7,8). Seasonality and epidemic clustering remain controversial (2,9). The etiologic role of the herpes simplex virus 1 (HSV-1) has been hypothesized since the work of McCormick in 1972 (10), while further studies from Murakami (1996), who showed evidence of HSV-1 reactivation in the geniculate ganglia (11), supported this theory. However, causality was never proven and the pattern of presentation of Bell’s palsy does not match that of other diseases caused by HSV-1 (2,12). Pathologic mechanisms also remain unknown (7). Facial nerve inflammation and edema are accepted and are the logical basis for surgical decompression procedures performed in some patients (7,12); however, the exact pathways implicated in neural dysfunction are under discussion (2). A recent paper suggested that acute axonal degeneration in response to a viral infection may be an innate immune response to prevent virus propagation to the central nervous system (2,13).There is no diagnostic marker for Bell’s palsy and it remains a clinical diagnosis and an exclusion diagnosis, as implied by the idiopathic label (7).

## Materials and Methods

We conducted a retrospective cross-sectional study to review demographic characteristics of our patients and the medical care administered in our emergency department. We included emergency episodes occurring between 1 January 2012 and 31 December 2015 and codified as facial nerve pathology. Duplicates, codifying errors and episodes related to patients with previous PFP seeking medical care for other reasons were excluded. IBM  SPSS™ software was used for statistical analysis; P<0,005 was considered for statistical significance. A χ^2^ test was used to study categorical variables and a binary logistic regression was used to study the probability of early imaging study.

## Results

In total, 582 emergency episodes were obtained. Seventy-six episodes met the exclusion criteria, leaving a final number of 506 cases of PFP. The most common diagnosis was idiopathic PFP–Bell’s palsy(n= 495; 97.8%). Less frequent diagnoses were Ramsay-Hunt syndrome (0.8%), PFP secondary to an infectious otologic pathology (0.8%), and PFP caused by neoplastic disease (0.6%).

We observed a higher percentage of Bell’s palsy diagnosis than expected (97.8% vs ~70%) (5,6). The authors considered several explanations for this; the emergency environment does not allow exhaustive investigation to exclude other diagnoses and therefore, in some cases, a diagnosis of Bell’s palsy in our patients could have been assumptive rather than exclusive. Initial presentation of disease, masked viral infection (zoster sin herpete), and non-universal observation by a specialist otolaryngologist may also be possible explanations.

Due to inexpressive representation of other causes of PFP in our study, we focused our analyses on the 495 patients who were considered to have idiopathic PFP (Table.1).

**Table 1:** Demographic, clinical and therapeutic management in patients with Bell’s palsy

Variable		n /total (%)
Age (years/mean ± SD)		46,87 ± 21,49
Gender	MaleFemale	248 (50,1) 247 (49,9)
Race	Caucasian Black Missing	469 (98,9) 5 (1,1) 21
Time of presentation	< 24h 24-48h 48-72h >72h Missing	307 (68,9) 73 (16,6) 37 (8,4) 23 (5,2) 55
Symptoms		
Sensitive Dysgeusia Cochleovestibular Xerophthalmia Neurological		234 /436 (53,7) 22 /438 (5) 30 /440 (6,8) 1 /438 (0,2) 49 /441 (11,1)
Pregnancy		4 /227 (1,8)
PUAI		45 /420 (10,7)
Hypertension		145 /402 (36,1)
Diabetes mellitus 2		69 /402 (17,2)
HIV		3 /402 (0,7)
Autoimmune disease		9 /402 (2,2)
Previous PFP		50 /412 (12,1)
CT scan <72h		157 /495 (31,7)
Laboratory tests		132 /494 (26,7)
PFP severity	HB 1 HB 2 HB 3 HB 4 HB 5 HB 6 Missing	12 (3,9) 94 (30,6) 117 (38,1) 67 (21,8) 17 (5,5) 0 188
Referred to	General physician Neurology Otolaryngology Other	336 (67,9) 58 (11,7) 96 (19,4) 5 (1)
Otolaryngology observation		326 /495 (65,9)
Drug therapy	None SS AV SS + AV Other Missing	56 (11,7) 327 (68,3) 2 (0,4) 81 (16,9) 13 (2,7) 16

HB: House-Brackmann facial grading system; PUAI: Previous Upper Airway Infection; CT scan: Computerized tomography scanning; PFP: Peripheral Facial Palsy; SS: systemic steroids; AV: systemic antiviral

## Discussion

**Fig 1:**
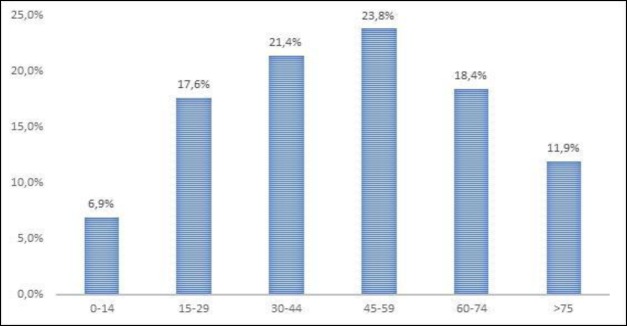
Age distribution of Bell’s palsy

**Fig 2:**
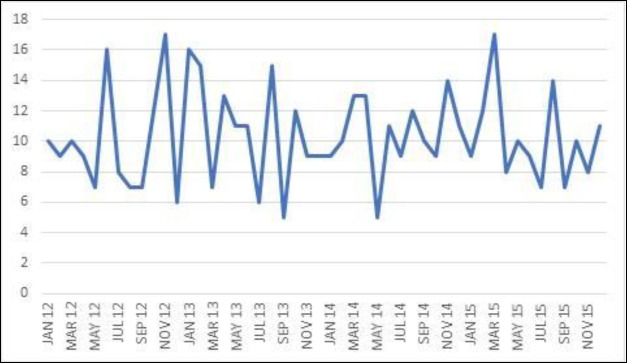
Number of cases of Bell’s palsy per month of study

**Fig 3:**
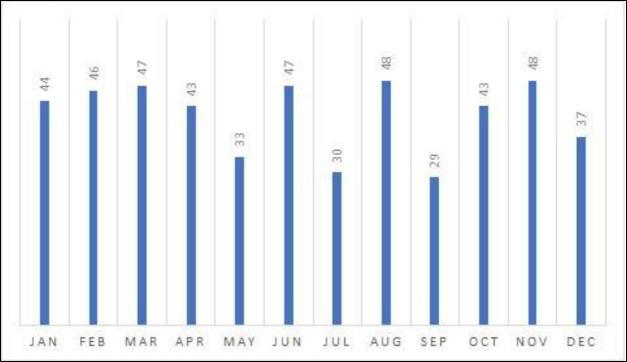
Bell’s palsy cases grouped per month

Our hospital emergency department is the reference medical unit for some 334,000 people, representing 38 cases/100,000/year. There was equal distribution among genders (50.6% females) and, as expected (2,7), all age ranges were affected (1 to 102 years, $\bar{\chi }=46,87\pm 21,49$), with a peak incidence in the 45–59 year age group (Fig.1). No clear epidemic clustering was observed (Fig.2), nor any other epidemic phenomena favoring seasonality (Fig.3).

Arterial hypertension was the most prevalent comorbidity in our study (36.1%), followed by diabetes mellitus 2 (17.2%), previous upper airways infection (PUAI) (10.7%), autoimmune disease (2.2%) and human immunodeficiency virus (HIV) infection (0.7%). In total, 1.8% of women were pregnant. Since this study was not designed with a control group, it was not possible to define which of these comorbidities are risk factors for Bell’s palsy. However, the authors consulted the EuroHeart II study (14), a comprehensive evaluation of cardiovascular disease in the European population, and reviewed the information on arterial hypertension given by this paper. Data from the Portuguese National Census were used to estimate Portuguese total population (15). The authors concluded that hypertension is not a statistically significant risk factor for Bell’s palsy in the study population (44.1% vs 41.9%, P=0.308, in patients over 25 years). Despite the limitations of our conclusion, the results are concordant with some recent studies which found no independent role for hypertension in patients younger than 40 years, and only a mild association in older patients (8).

A similar procedure was undertaken to investigate diabetes mellitus as a risk factor. In this instance, the authors used data from the Portuguese national observatory (16).

A statistically significant difference was found between the Bell’s palsy population and the general population (17.8% vs 7.4%, P<0.001, patients aged 20 to 79 years). Again, clear limitations are recognized, but the results are in accordance with most papers which recognize diabetes mellitus 2 as an important risk factor for Bell’s palsy (6–8).

PUAI was reported more frequently in the pediatric population (34.7% vs 7.5%, P<0.001). While in adults, idiopathic PFP is the final diagnosis in 70% of cases (5,6), in children it represents a smaller proportion (40–50%), with infectious and traumatic causes accounting for a higher proportion of cases than in adults (17–20). It is not clear whether pediatric idiopathic PFP is a variant disease in which infection is a causative agent or if the association is circumstantial because of the immaturity of the immune system of children that makes them more susceptible to infectious disease (18). 

From the authors’ understanding, there are no studies suggesting a pathophysiological difference between pediatric and adult idiopathic PFP. Our study does not allow further conclusions, however, since further work is needed. Most patients sought medical care in the early stages of presentation: 69.8% in the initial 24h, 16.6% at 24–48h, 8.4% at 48–72h, and only 5.2% after 72h of evolution. From the authors’ point of view, this is a consequence of the pattern of presentation of the disease of facial dysmorphia, and also because of patients fear of the more well-known (and more debilitating) central vascular events. This allows the physician to initiate early treatment (within the first 72h), when it is more effective in the majority of patients(7). A considerable proportion of patients (38.4%) return to the emergency department complaining of isolated facial weakness. When additional symptoms are reported, sensitive alterations of the head, neck and oral cavity (pain, hypoesthesia, hyperesthesia, numbness) are the most common (53.7%) symptomatic complex. Neurological symptoms, such as diplopia, peripheric muscular weakness or dysarthria, are reported in 11.8% of patients; cochleovestibular symptoms, such as vertigo, hypoacusia and tinnitus are present in a minority 6.8%; while dysgeusia (5.0%) and xerophthalmia (0.2%) are even less common complaints. In our hospital, the otolaryngologist is present in the emergency department for only 12h each day (8h to 20h). As such, more than two-thirds of patients who seek medical care are evaluated in the first instance by a general physician, or a general pediatrician when the patient is younger than 18 years, while only one out of five is initially observed by an otolaryngologist. Despite this, more than a half of those not initially referred to an otolaryngologist were evaluated by this specialty during the emergency episode.

**Fig 4:**
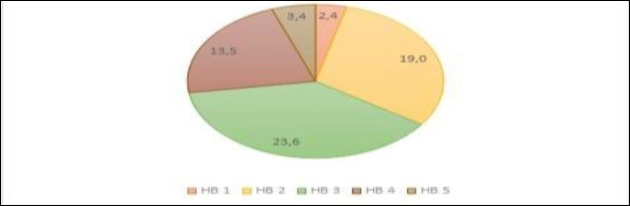
PFP severity on presentation (House-Brackmann Grading System

In most cases, Bell’s palsy is a benign condition with good rates of recovery (3,4). In this study, among the 62% classified according to the House-Brackmann scale (21), the majority (72.6%) had mild-to-moderate symptoms(HB≤3) (Fig.4). Since this is a cross-sectional observational study, there are no data regarding rate of recovery.

Considering in-hospital medical care, 31.7% patients were subject to early (<72h) imaging (computed tomography [CT] scan) and 26.7% to laboratory studies. According to the American Academy of Otolaryngology– Head and Neck Surgery Guidelines on Bell’s palsy, routine imaging and laboratory studies are discouraged (7). In terms of an explanation for the higher number of patients exposed to CT scans, there was a statistically significant difference in the rate of CT scan requests among specialties, with otolaryngology having the lower rate (8.3%) and general physicians having the highest (41.7%). Neurology ranked in the middle (15.5%). It was assumed that patients referred to general physicians were more exposed to comorbidities –such as arterial hypertension and diabetes mellitus–and reported more alarming neurological symptoms that made a central event more likely in this group that would likewise justify CT scan requisition. A binary logistic regression model was undertaken to justify this difference. Surprisingly, the odds ratio of the Cox & Snell R square test was 0.545. Thus, despite the extensive list of evaluated variables, only a small proportion of this difference was justified (Table.2).

**Table 2:** Binary logistic regression model for CT scan ordering

**Variables in the equation**							
		**B**	**S.E.**	**Wald**	**df**	**Sig.**	**Exp(B)**
Step 1^a^	Gender	-1,303	0,588	4,917	1	0,027	0,272
	Age	-0,013	0,016	0,636	1	0,425	0,987
	Race	0,109	9,413	0,000	1	0,991	1,115
	Referred to	0,561	0,440	1,628	1	0,202	1,753
	Time of presentation	-0,287	0,369	0,607	1	0,436	0,750
	Sensitive symptoms	0,687	0,575	1,429	1	0,232	1,989
	Dysgeusia	1,125	1,226	0,843	1	0,359	3,082
	Cochleovestibular symptoms	3,292	0,995	10,950	1	0,001	26,903
	Neurologic symptoms	2,695	0,975	7,637	1	0,006	14,808
	PUAI	-2,276	1,235	3,396	1	0,065	0,103
	Hypertension	0,571	0,716	0,635	1	0,425	1,769
	DM II	0,452	1,023	0,195	1	0,659	1,571
	Comorbidities	-0,327	0,668	0,240	1	0,624	0,721
	Previous PFP episode	0,358	1,083	0,109	1	0,741	1,431
	Lab test	5,031	0,735	46,892	1	0,000	153,114
	Otolaryngology observation	-0,496	0,867	0,327	1	0,567	0,609
	House−Brackmann Grading	0,219	0,299	0,538	1	0,463	1,245
	Constant	-3,108	1,430	4,725	1	0,030	0,045

a . Variable(s) entered on step 1: Gender. Age. Race. Referred to. Time of presentation. Sensitive symptoms. Dysgeusia. Cochleovestibular symptoms. Neurologic symptoms. Pregnancy. PUAI (Previous Upper Airway Infection). Hypertension. DM II (Diabetes mellitus 2). Comorbidities. Previous PFP (Peripheral Facial Palsy) episode. Lab (Laboratory) test. Otolaryngology observation. House−Brackmann Grading.

This study was not designed to explain this difference among specialties, and therefore no definitive explanation is attempted. However, the inclusion of patients suffering from an episode of PFP in stroke management protocols may partially justify this fact. Lack of knowledge about this disease and inherent fear of underdiagnosing a potential life-threatening event (stroke) may also sustain the practice of over-defensive medicine.

Excessive reliance on the diagnostic sensitivity of CT scans may also be a reason for this difference. Moreover, current evidence strongly suggests that CT scan sensitivity in the early stages of a stroke is unacceptably low, and is no better than clinical observation (22,23).

**Fig 5:**
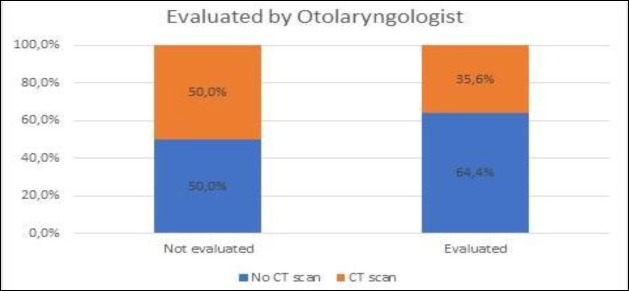
Influence of otolaryngologist observation in CT scan ordering in patients referred to general physician

The authors also compared the likelihood of undertaking a CT scan among patients not referred initially to otolaryngology. Those not initially referred but evaluated by this specialty during the emergency episode had a lower probability of receiving a CT scan than those never observed by otolaryngology (Fig.5). This tendency, however, did not reach statistical significance.

**Fig 6:**
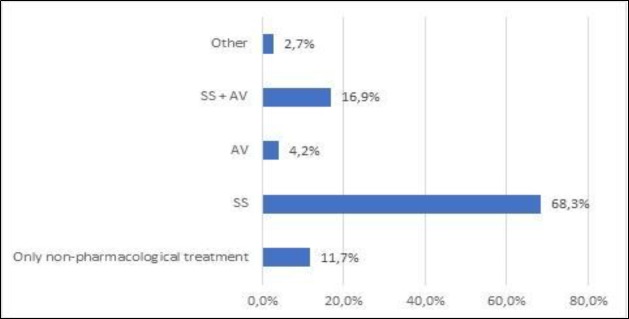
Drug therapy in Bell’s palsy patients; SS: systemic steroids; AV: systemic antiviral

Regarding pharmacological treatment after discharge, most patients received systemic glucocorticoids alone or in combination with other drugs (Fig.6). 

From the authors’ perspective, and according to American Guidelines (7), this represents good medical care. We found no association between PFP severity and drug therapy (Pearson χ^2^ test =28.065, P= 0.31). Despite not being evaluated in this study, and therefore no objective data being provided, most patients were advised to perform physical rehabilitation and eye care when needed. Evidence regarding the benefit of this is controversial (6,7,12), but it is clinically rooted advice. Considerations of surgical intervention are not discussed in this paper; no surgery was performed in any patient, and indeed there is a current trend away from this kind of procedure (6,7), despite success in very selected cases (2,19,24).

***Limitations***

This a retrospective, non-randomized study, and is therefore subject to several limitations. Some variables were intentionally omitted, such as side of palsy (sides were equally affected in most studies) and race (most of our population was Caucasian, with only five black patients and no Asian patients). There is an obvious selection bias since we searched for emergency episodes codified as facial nerve pathology. Patients suffering from Ramsay-Hunt syndrome may have be codified as suffering from otitis externa, while patients with neoplastic PFP may have been codified with parotid or facial nerve neoplasm. This would explain the disproportion between idiopathic and other forms of PFP observed in this study. There is also an observational bias in this study, in that the records were prepared by several physicians working in this hospital, with different differentiation backgrounds and a variable understanding of facial nerve pathology. This may make some physicians more likely to record some symptoms and signs than others. Another major limitation in this study is the lack of a control group. This is being considered for a following study, which may confirm some tendencies found in this paper.

## Conclusion

The present study does not allow clarification of the role of hypertension as a risk factor for Bell’s palsy. However, despite obvious limitations in this study, the authors conclude that our data support current evidence questioning the role of hypertension as an independent risk factor (8). Further investigation is needed on this matter.

The epidemiologic findings are consistent with most literature on Bell’s palsy (2,7). This validates the study findings and allows international guidelines on Bell’s palsy to be applied in the study population. There was a clear association between PUAI and pediatric age. Further studies to evaluate this association are needed.

Bell’s palsy is mostly a benign condition. This study does not allow evaluation of recovery (nor comparison among different treatment modalities) but considering current evidence (3,7), good rates are expected.

Drug therapy is widely used and follows current guidelines (7). Despite evidence of good medical practice, in-hospital orientation of Bell’s palsy patients deserves reflection. In fact, the disproportionate requisition of CT scans in the early stages of disease is potentially harmful in a dichotomic way: it exposes the patient to unnecessary radiation and gives the physician a false sense of security that may allow more life-threatening conditions to remain undiagnosed. Better bedside assessment (22) and an understanding of the benign course of the disease in most cases (3) is crucial for lowering the economic and iatrogenic burden of CT scans in emergency departments. The higher rates of CT scans ordered by non-ENT physicians reflect the singular role that otorhinolaryngologists have in managing Bell’s palsy patients; the lowering effect on CT scan requests not only has economic implications but also medical benefits, reducing the amount of radiation that these patients are exposed to.

## References

[1] Spencer C, Irving R (2016). Causes and management of facial nerve palsy. Br J Hosp Med.

[2] Eviston T, Croxson G, Kennedy P, Hadlock T, Krishnan A (2015). Bell’s palsy: aetiology, clinical features and multidisciplinary care. J Neurol Neurosurg Psychiatry.

[3] Ferreira M, Firmino-Machado J, Marques E, Santos P, Simões A, Duarte J (2016). Prognostic factors for recovery in Portuguese patients with Bell’s palsy. Neurol Res.

[4] Finsterer J (2008). Management of peripheral facial nerve palsy. Eur Arch Oto-Rhino-Laryngology.

[5] Monini S, Lazzarino A, Iacolucci C, Buffoni A, Barbara M (2010). Epidemiology of Bell’s palsy in an Italian Health District: incidence and case-control study. Acta Otorhinolaryngol Ital.

[6] Newadkar U, Chaudhari L, Khalekar Y (2016). Facial palsy, a disorder belonging to influential neurological dynasty: Review of literature. N Am J Med Sci.

[7] Baugh R, Basura G, Ishii L, Schwartz SR, Drumheller CM, Burkholder R (2013). Clinical Practice Guideline. Otolaryngol Neck Surg.

[8] Savadi-Oskouei D, Abedi A, Sadeghi-Bazargani H (2008). Independent role of hypertension in Bell’s palsy: A case-control study. Eur Neurol.

[9] Rowlands S, Hooper R, Hughes R, Burney P (2002). The epidemiology and treatment of Bell’s palsy in the UK. Eur J Neurol.

[10] Konrad-Martin D, Reavis KM, McMillan G, Helt WJ, Dille M (2014). Proposed comprehensive ototoxicity monitoring program for VA healthcare (COMP-VA). J Rehabil Res Dev.

[11] Murakami S, Mirzobuchi M, Nakashiro Y, Doi T, Hato N, Yanagihara N (1996). Bell’s palsy and herpes simplex virus: identification of viral DNA in endoneural fluid and muscle. Ann Int Med.

[12] Glass G, Tzafetta K (2014). Bell’s palsy: A summary of current evidence and referral algorithm. Fam Pract.

[13] Galluzzi L, Blomgren K, Kroemer G (2009). Mitochondrial membrane permeabilization in neuronal injury. Nat Rev Neurosci.

[14] Nichols M, Townsend N, Scarborough P, Rayner M, Leal J, Luengo-Fernandez R European Cardiovascular Disease Statistics 2012.

[15] Instituto Nacional de Estatística IP Censos 2011 Resultados Definitivos - Portugal 2011.

[16] Sociedade Portuguesa de Diabetologia. Diabetes: Factos E Números – O Ano de 2014 − Relatório Anual Do Observatório Nacional Da Diabetes.

[17] Ciorba A, Corazzi V, Conz V, Bianchini C, Aimoni C, Facial nerve paralysis in children (2015). World J Clin Cases.

[18] Wang C, Chang Y, Shih H, Chen C, Chen J (2010). Facial Palsy in Children. Pediatr Emerg Care.

[19] Reddy S, Redett R (2015). Facial paralysis in children. Facial Plast Surg.

[20] Evans A, Licameli G, Brietzke S, Whittemore K, Kenna M (2005). Pediatric facial nerve paralysis: Patients, management and outcomes. Int J Pediatr Otorhinolaryngol.

[21] House J, Brackmann D (1985). Facial nerve grading system. Otolaryngol Head Neck Surg.

[22] Kattah J, Talkad A, Wang D, Hsieh Y, Newman-Toker D (2009). HINTS to diagnose stroke in the acute vestibular syndrome: Three-step bedside oculomotor examination more sensitive than early MRI diffusion-weighted imaging. Stroke.

[23] Chalela J, Kidwell C, Nentwich L, Luby M, Butman JA, Demchuk AM (2007). Magnetic resonance imaging and computed tomography in emergency assessment of patients with suspected acute stroke: a prospective comparison. Lancet.

[24] Kim C, Lelli G (2013). Current considerations in the management of facial nerve palsy. Curr Opin Ophthalmol.

